# *N*′-[(2*Z*)-4-Oxo-4-phenyl­but-2-en-2-yl]pyridine-4-carbohydrazide

**DOI:** 10.1107/S1600536812000529

**Published:** 2012-01-14

**Authors:** Rahman Bikas, Parisa Mahboubi Anarjan, Sanam Aslekhademi, Seik Weng Ng, Edward R. T. Tiekink

**Affiliations:** a Young Researchers Club, Tabriz Branch, Islamic Azad University, Tabriz, Iran; b Department of Chemistry, University of Zanjan, 45195-313 Zanjan, Iran; c Department of Chemistry, Faculty of Science, Yasouj University, Yasouj, Iran; d Department of Chemistry, University of Malaya, 50603 Kuala Lumpur, Malaysia; e Chemistry Department, Faculty of Science, King Abdulaziz University, PO Box 80203 Jeddah, Saudi Arabia

## Abstract

There are significant twists in the title compound, C_16_H_15_N_3_O_2_, as seen in the dihedral angle between the benzene and adjacent but-2-enal group [29.26 (4)°] and between the pyridine ring and amide group [24.79 (6)°]. A twist is also evident around the hydrazine bond [the C—N—N—C torsion angle is −138.25 (13)°]. The conformation about the ethene bond is *Z*. An intra­molecular N—H⋯O hydrogen bond involving the benzoyl O atom and leading to an *S*(6) motif is formed. Significant delocalization of π-electron density is found in this part of the mol­ecule. In the crystal, helical supra­molecular chains aligned along the *b* axis and mediated by N—H⋯O hydrogen bonds are formed.

## Related literature

For the structures of related carbohydrazides, see: Bikas *et al.* (2010 ▶, 2012 ▶).
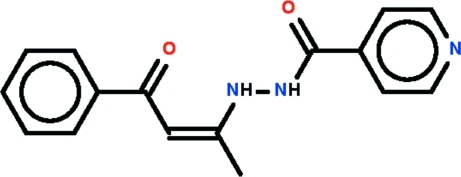


## Experimental

### 

#### Crystal data

C_16_H_15_N_3_O_2_
*M* *_r_* = 281.31
Monoclinic, 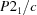

*a* = 15.7640 (4) Å
*b* = 6.5194 (1) Å
*c* = 13.3093 (3) Å
β = 93.579 (2)°
*V* = 1365.15 (5) Å^3^
*Z* = 4
Cu *K*α radiation
μ = 0.76 mm^−1^
*T* = 100 K
0.20 × 0.10 × 0.05 mm

#### Data collection

Agilent SuperNova Dual diffractometer with an Atlas detector
Absorption correction: multi-scan (*CrysAlis PRO*; Agilent, 2010 ▶) *T*_min_ = 0.864, *T*_max_ = 0.963
5321 measured reflections
2808 independent reflections
2397 reflections with *I* > 2σ(*I*)
*R*_int_ = 0.022

#### Refinement

*R*[*F* ^2^ > 2σ(*F* ^2^)] = 0.039
*wR*(*F* ^2^) = 0.109
*S* = 1.02
2808 reflections
199 parameters
H atoms treated by a mixture of independent and constrained refinement
Δρ_max_ = 0.30 e Å^−3^
Δρ_min_ = −0.23 e Å^−3^

### 

Data collection: *CrysAlis PRO* (Agilent, 2010 ▶); cell refinement: *CrysAlis PRO*; data reduction: *CrysAlis PRO*; program(s) used to solve structure: *SHELXS97* (Sheldrick, 2008 ▶); program(s) used to refine structure: *SHELXL97* (Sheldrick, 2008 ▶); molecular graphics: *X-SEED* (Barbour, 2001 ▶); software used to prepare material for publication: *publCIF* (Westrip, 2010 ▶).

## Supplementary Material

Crystal structure: contains datablock(s) global, I. DOI: 10.1107/S1600536812000529/hg5158sup1.cif

Structure factors: contains datablock(s) I. DOI: 10.1107/S1600536812000529/hg5158Isup2.hkl

Supplementary material file. DOI: 10.1107/S1600536812000529/hg5158Isup3.cml

Additional supplementary materials:  crystallographic information; 3D view; checkCIF report

## Figures and Tables

**Table 1:** Hydrogen-bond geometry (Å, °)

*D*—H⋯*A*	*D*—H	H⋯*A*	*D*⋯*A*	*D*—H⋯*A*
N2—H2⋯O2^i^	0.88 (2)	1.90 (2)	2.750 (2)	163 (2)
N3—H3⋯O2	0.90 (2)	1.91 (2)	2.607 (1)	133 (2)

Symmetry code: (i) 
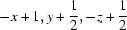
.

**Fig. 1.**
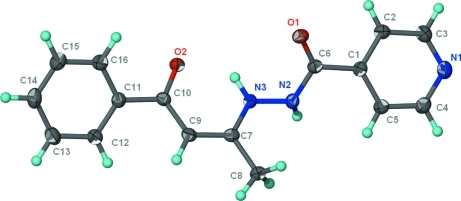
Molecular structure of (I) with displacement ellipsoids at the 70% probability level; hydrogen atoms are drawn as spheres of arbitrary radius.

**Fig. 2.**
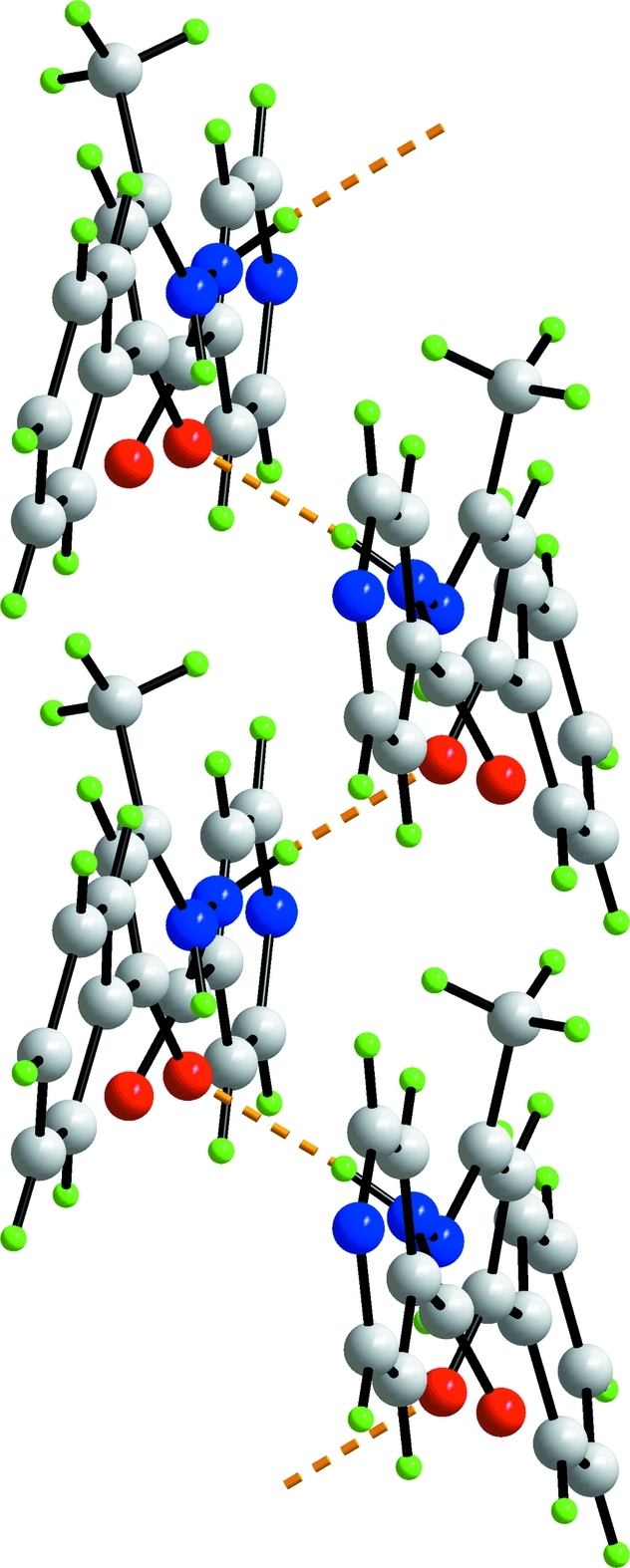
Supramolecular helical chain parallel to [010] in (I). The N—H···O hydrogen bonds are shown as orange dashed lines.

## supplementary crystallographic information

### Comment

The reaction of acid hydrazides (R—C(═ O)–NH–NH_2_) with β-diketones forms a class of molecules that can function as tridentate Schiff base ligands and which can have diverse tautomeric states. As part of continuing studies on the synthesis and characterization of aroylhydrazone compounds (Bikas *et al.*, 2010; Bikas *et al.*, 2012), we describe herein the crystal structure of (*Z*)-*N*'-(4-oxo-4-phenylbut-2-en-2-yl)isonicotinohydrazide, (I).

The structure determination of (I), Fig. 1, shows that the molecule exists in the di-enone form and that the conformation about the ethene bond is *Z*. However, it is noted that the ketone C═O bond length of 1.2705 (15) Å is significantly longer than the amide C═O bond length of 1.2213 (16) Å. Further, the formally ethene double bond length of 1.3905 (18) Å is only marginally longer than the C(═O)—C-ethene bond of 1.4097 (18) Å. These observations coupled with the shorter than expected N3—C7 bond length of 1.3369 (16) Å and the planarity of this residue (the r.m.s. = 0.0141 Å, including the N—H atom) indicates significant delocalization of π-electron density over the non-H atoms. It is noted that in this residue a six-membered ring is formed through the agency of an intramolecular N—H···O hydrogen bond, Table 1.

There are significant twists in the molecule with the benzene group twisted out of the plane through the adjacent but-2-enal group (dihedral angle = 29.26 (4)°) and the pyridyl ring twisted out of the plane through the amide group (dihedral angle = 24.79 (6)°). There is also a twist around the hydrazine bond as seen in the value of the C6—N2—N3—C7 torsion angle of -138.25 (13)°.

The most prominent feature of the crystal packing is the formation of helical supramolecular chains along [010] mediated by N—H···O hydrogen bonds, Fig. 2 and Table 1.

### Experimental

All reagents were commercially available and used as received. A methanol (10 ml) solution of benzoylacetone (1.5 mmol) was added drop-wise to a methanol solution (10 ml) of 4-pyridinecarboxylic acid hydrazide (1.5 mmol), and the mixture was refluxed for 3 h. Then the solution was evaporated on a steam bath to 5 ml and cooled to room temperature. Light-yellow precipitates of the title compound were separated and filtered off, washed with 3 ml of cooled methanol and then dried in air. Crystals of the title compound were obtained from its methanol solution by slow solvent evaporation. Yield 92%. Selected IR (cm^-1^): 3155 (s, broad), 1690 (*versus*), 1596 (*s*), 1520 (*m*), 1309 (*s*), 1224 (*s*), 931 (*versus*), 772 (*s*).

### Refinement

Carbon-bound H-atoms were placed in calculated positions [C—H 0.95 to 0.98 Å, *U*_iso_(H) 1.2 to 1.5*U*_eq_(C)] and were included in the refinement in the riding model approximation. The amino H-atoms were located in a difference Fourier map, and were refined with a distance restraint of N–H 0.88±0.01 Å; their *U*_iso_ values were refined.

### Crystal data

**Table:**

C_16_H_15_N_3_O_2_	*F*(000) = 592
*M**_r_* = 281.31	*D*_x_ = 1.369 Mg m^−^^3^
Monoclinic, *P*2_1_/*c*	Cu *K*α radiation, λ = 1.54184 Å
Hall symbol: -P 2ybc	Cell parameters from 2202 reflections
*a* = 15.7640 (4) Å	θ = 2.8–76.4°
*b* = 6.5194 (1) Å	µ = 0.76 mm^−^^1^
*c* = 13.3093 (3) Å	*T* = 100 K
β = 93.579 (2)°	Prism, colourless
*V* = 1365.15 (5) Å^3^	0.20 × 0.10 × 0.05 mm
*Z* = 4	

### Data collection

**Table:**

Agilent SuperNova Dual diffractometer with an Atlas detector	2808 independent reflections
Radiation source: SuperNova (Cu) X-ray Source	2397 reflections with *I* > 2σ(*I*)
Mirror	*R*_int_ = 0.022
Detector resolution: 10.4041 pixels mm^-1^	θ_max_ = 76.6°, θ_min_ = 2.8°
ω scan	*h* = −14→19
Absorption correction: multi-scan (CrysAlis PRO; Agilent, 2010)	*k* = −8→4
*T*_min_ = 0.864, *T*_max_ = 0.963	*l* = −13→16
5321 measured reflections	

### Refinement

**Table:**

Refinement on *F*^2^	Primary atom site location: structure-invariant direct methods
Least-squares matrix: full	Secondary atom site location: difference Fourier map
*R*[*F*^2^ > 2σ(*F*^2^)] = 0.039	Hydrogen site location: inferred from neighbouring sites
*wR*(*F*^2^) = 0.109	H atoms treated by a mixture of independent and constrained refinement
*S* = 1.02	*w* = 1/[σ^2^(*F*_o_^2^) + (0.0568*P*)^2^ + 0.4474*P*] where *P* = (*F*_o_^2^ + 2*F*_c_^2^)/3
2808 reflections	(Δ/σ)_max_ = 0.001
199 parameters	Δρ_max_ = 0.30 e Å^−^^3^
0 restraints	Δρ_min_ = −0.23 e Å^−^^3^

### Fractional atomic coordinates and isotropic or equivalent isotropic displacement parameters (Å^2^)

**Table:**

	*x*	*y*	*z*	*U*_iso_*/*U*_eq_	
O1	0.64472 (6)	0.16395 (15)	0.10581 (7)	0.0222 (2)	
O2	0.39259 (6)	0.19577 (14)	0.15136 (7)	0.0192 (2)	
N1	0.93135 (7)	0.45503 (19)	0.21632 (9)	0.0244 (3)	
N2	0.61169 (7)	0.47152 (17)	0.17399 (9)	0.0182 (2)	
N3	0.52548 (7)	0.43426 (17)	0.15465 (8)	0.0176 (2)	
C1	0.75840 (8)	0.3754 (2)	0.17323 (9)	0.0170 (3)	
C2	0.81664 (9)	0.2161 (2)	0.18446 (10)	0.0215 (3)	
H2A	0.7986	0.0775	0.1773	0.026*	
C3	0.90178 (9)	0.2629 (2)	0.20629 (11)	0.0249 (3)	
H3A	0.9411	0.1529	0.2145	0.030*	
C4	0.87448 (9)	0.6070 (2)	0.20366 (10)	0.0225 (3)	
H4	0.8945	0.7442	0.2095	0.027*	
C5	0.78809 (8)	0.5759 (2)	0.18252 (10)	0.0194 (3)	
H5	0.7502	0.6888	0.1746	0.023*	
C6	0.66681 (8)	0.3232 (2)	0.14810 (9)	0.0168 (3)	
C7	0.47294 (8)	0.58056 (19)	0.11764 (9)	0.0170 (3)	
C8	0.50980 (8)	0.7859 (2)	0.09507 (10)	0.0197 (3)	
H8A	0.5348	0.8469	0.1574	0.030*	
H8B	0.4648	0.8758	0.0661	0.030*	
H8C	0.5539	0.7695	0.0470	0.030*	
C9	0.38667 (8)	0.5408 (2)	0.09966 (10)	0.0174 (3)	
H9	0.3508	0.6492	0.0753	0.021*	
C10	0.34988 (8)	0.3474 (2)	0.11585 (9)	0.0166 (3)	
C11	0.25751 (8)	0.3125 (2)	0.08761 (9)	0.0173 (3)	
C12	0.19729 (8)	0.4687 (2)	0.08987 (10)	0.0204 (3)	
H12	0.2147	0.6046	0.1066	0.025*	
C13	0.11164 (9)	0.4262 (2)	0.06766 (11)	0.0251 (3)	
H13	0.0706	0.5322	0.0711	0.030*	
C14	0.08620 (9)	0.2289 (2)	0.04045 (11)	0.0260 (3)	
H14	0.0277	0.2003	0.0250	0.031*	
C15	0.14599 (9)	0.0735 (2)	0.03576 (11)	0.0248 (3)	
H15	0.1286	−0.0610	0.0163	0.030*	
C16	0.23130 (9)	0.1148 (2)	0.05958 (10)	0.0209 (3)	
H16	0.2721	0.0080	0.0568	0.025*	
H2	0.6210 (11)	0.549 (3)	0.2275 (14)	0.029 (5)*	
H3	0.5045 (11)	0.308 (3)	0.1636 (13)	0.028 (4)*	

### Atomic displacement parameters (Å^2^)

**Table:**

	*U*^11^	*U*^22^	*U*^33^	*U*^12^	*U*^13^	*U*^23^
O1	0.0214 (5)	0.0160 (5)	0.0288 (5)	−0.0010 (4)	−0.0021 (4)	−0.0039 (4)
O2	0.0180 (4)	0.0157 (5)	0.0239 (5)	0.0014 (3)	0.0007 (4)	0.0039 (4)
N1	0.0184 (6)	0.0260 (6)	0.0289 (6)	−0.0015 (5)	0.0014 (5)	−0.0019 (5)
N2	0.0150 (5)	0.0182 (5)	0.0213 (6)	−0.0014 (4)	−0.0003 (4)	−0.0038 (4)
N3	0.0146 (5)	0.0158 (5)	0.0224 (5)	−0.0007 (4)	0.0006 (4)	0.0004 (4)
C1	0.0171 (6)	0.0179 (6)	0.0159 (6)	−0.0012 (5)	0.0009 (5)	−0.0004 (5)
C2	0.0205 (6)	0.0171 (6)	0.0268 (7)	0.0007 (5)	0.0009 (5)	0.0007 (5)
C3	0.0198 (7)	0.0235 (7)	0.0312 (7)	0.0024 (5)	0.0006 (6)	0.0009 (6)
C4	0.0219 (7)	0.0197 (7)	0.0261 (7)	−0.0038 (5)	0.0028 (5)	−0.0020 (5)
C5	0.0198 (6)	0.0173 (6)	0.0214 (6)	−0.0004 (5)	0.0024 (5)	−0.0006 (5)
C6	0.0183 (6)	0.0155 (6)	0.0165 (6)	−0.0001 (5)	0.0011 (5)	0.0017 (5)
C7	0.0204 (6)	0.0147 (6)	0.0159 (6)	0.0009 (5)	0.0021 (5)	−0.0006 (5)
C8	0.0210 (6)	0.0152 (6)	0.0231 (6)	−0.0009 (5)	0.0017 (5)	0.0008 (5)
C9	0.0180 (6)	0.0160 (6)	0.0183 (6)	0.0022 (5)	0.0015 (5)	0.0010 (5)
C10	0.0177 (6)	0.0163 (6)	0.0160 (6)	0.0024 (5)	0.0030 (5)	0.0007 (5)
C11	0.0168 (6)	0.0189 (6)	0.0162 (6)	−0.0006 (5)	0.0016 (5)	0.0016 (5)
C12	0.0193 (6)	0.0194 (6)	0.0227 (6)	0.0013 (5)	0.0019 (5)	0.0009 (5)
C13	0.0189 (7)	0.0277 (7)	0.0289 (7)	0.0043 (5)	0.0026 (5)	0.0020 (6)
C14	0.0170 (6)	0.0323 (8)	0.0284 (7)	−0.0037 (5)	−0.0012 (5)	0.0018 (6)
C15	0.0237 (7)	0.0233 (7)	0.0271 (7)	−0.0050 (5)	0.0003 (5)	−0.0014 (6)
C16	0.0209 (7)	0.0196 (7)	0.0223 (6)	0.0003 (5)	0.0024 (5)	0.0000 (5)

### Geometric parameters (Å, °)

**Table:**

O1—C6	1.2213 (16)	C7—C8	1.4973 (17)
O2—C10	1.2705 (15)	C8—H8A	0.9800
N1—C4	1.3395 (18)	C8—H8B	0.9800
N1—C3	1.3401 (18)	C8—H8C	0.9800
N2—C6	1.3587 (17)	C9—C10	1.4097 (18)
N2—N3	1.3887 (15)	C9—H9	0.9500
N2—H2	0.878 (18)	C10—C11	1.4984 (17)
N3—C7	1.3369 (16)	C11—C12	1.3938 (18)
N3—H3	0.899 (18)	C11—C16	1.3974 (19)
C1—C2	1.3884 (18)	C12—C13	1.3918 (18)
C1—C5	1.3914 (18)	C12—H12	0.9500
C1—C6	1.5006 (17)	C13—C14	1.389 (2)
C2—C3	1.3894 (19)	C13—H13	0.9500
C2—H2A	0.9500	C14—C15	1.388 (2)
C3—H3A	0.9500	C14—H14	0.9500
C4—C5	1.3882 (19)	C15—C16	1.3887 (19)
C4—H4	0.9500	C15—H15	0.9500
C5—H5	0.9500	C16—H16	0.9500
C7—C9	1.3905 (18)		
			
C4—N1—C3	116.92 (12)	C7—C8—H8B	109.5
C6—N2—N3	117.55 (11)	H8A—C8—H8B	109.5
C6—N2—H2	122.5 (11)	C7—C8—H8C	109.5
N3—N2—H2	111.3 (11)	H8A—C8—H8C	109.5
C7—N3—N2	121.34 (11)	H8B—C8—H8C	109.5
C7—N3—H3	118.6 (11)	C7—C9—C10	123.22 (12)
N2—N3—H3	120.0 (11)	C7—C9—H9	118.4
C2—C1—C5	118.48 (12)	C10—C9—H9	118.4
C2—C1—C6	118.35 (12)	O2—C10—C9	122.62 (12)
C5—C1—C6	123.13 (12)	O2—C10—C11	117.35 (11)
C1—C2—C3	118.80 (13)	C9—C10—C11	120.00 (11)
C1—C2—H2A	120.6	C12—C11—C16	119.30 (12)
C3—C2—H2A	120.6	C12—C11—C10	122.43 (12)
N1—C3—C2	123.49 (13)	C16—C11—C10	118.26 (12)
N1—C3—H3A	118.3	C13—C12—C11	120.19 (13)
C2—C3—H3A	118.3	C13—C12—H12	119.9
N1—C4—C5	123.92 (13)	C11—C12—H12	119.9
N1—C4—H4	118.0	C14—C13—C12	120.03 (13)
C5—C4—H4	118.0	C14—C13—H13	120.0
C4—C5—C1	118.39 (12)	C12—C13—H13	120.0
C4—C5—H5	120.8	C15—C14—C13	120.13 (13)
C1—C5—H5	120.8	C15—C14—H14	119.9
O1—C6—N2	123.60 (12)	C13—C14—H14	119.9
O1—C6—C1	122.56 (12)	C16—C15—C14	119.93 (13)
N2—C6—C1	113.82 (11)	C16—C15—H15	120.0
N3—C7—C9	120.46 (12)	C14—C15—H15	120.0
N3—C7—C8	118.20 (11)	C15—C16—C11	120.38 (13)
C9—C7—C8	121.33 (11)	C15—C16—H16	119.8
C7—C8—H8A	109.5	C11—C16—H16	119.8
			
C6—N2—N3—C7	−138.25 (13)	N3—C7—C9—C10	−1.89 (19)
C5—C1—C2—C3	1.2 (2)	C8—C7—C9—C10	177.29 (12)
C6—C1—C2—C3	179.09 (12)	C7—C9—C10—O2	2.3 (2)
C4—N1—C3—C2	−0.4 (2)	C7—C9—C10—C11	−175.56 (11)
C1—C2—C3—N1	−0.6 (2)	O2—C10—C11—C12	151.66 (13)
C3—N1—C4—C5	0.9 (2)	C9—C10—C11—C12	−30.34 (18)
N1—C4—C5—C1	−0.4 (2)	O2—C10—C11—C16	−27.33 (17)
C2—C1—C5—C4	−0.68 (19)	C9—C10—C11—C16	150.67 (12)
C6—C1—C5—C4	−178.49 (12)	C16—C11—C12—C13	2.1 (2)
N3—N2—C6—O1	2.78 (19)	C10—C11—C12—C13	−176.86 (12)
N3—N2—C6—C1	−179.11 (10)	C11—C12—C13—C14	−1.8 (2)
C2—C1—C6—O1	−24.59 (19)	C12—C13—C14—C15	0.3 (2)
C5—C1—C6—O1	153.23 (13)	C13—C14—C15—C16	0.8 (2)
C2—C1—C6—N2	157.27 (12)	C14—C15—C16—C11	−0.5 (2)
C5—C1—C6—N2	−24.91 (18)	C12—C11—C16—C15	−1.0 (2)
N2—N3—C7—C9	−179.40 (11)	C10—C11—C16—C15	178.01 (12)
N2—N3—C7—C8	1.39 (18)		

### Hydrogen-bond geometry (Å, °)

**Table:**

*D*—H···*A*	*D*—H	H···*A*	*D*···*A*	*D*—H···*A*
N2—H2···O2^i^	0.88 (2)	1.90 (2)	2.750 (2)	163 (2)
N3—H3···O2	0.90 (2)	1.91 (2)	2.607 (1)	133 (2)

Symmetry codes: (i) −*x*+1, *y*+1/2, −*z*+1/2.
